# Paying for precision: funding approaches for N-of-1 trials of individualized gene targeted therapies

**DOI:** 10.1186/s13023-026-04388-1

**Published:** 2026-05-21

**Authors:** Nicole Nolen, Annemieke Aartsma-Rus, Christine Caneva, Curtis R. Coughlin II, Margot A. Cousin, Catherine Douthwright, Holm Graessner, Olivia Kim-McManus, Ashley Kuniholm, Stefanie Leonard, Andrea Martinsen, Margaret Meserve, Matthis Synofzik, Booma Yandava, Timothy W. Yu, Scott Demarest, Roger J. Paxton

**Affiliations:** 1 N=1 Collaborative, Somerville, MA 02143 USA; 2 Department of Human Genetics, Leiden University Medical Center, Leiden, The Netherlands; 3 Precision Medicine Institute, Children’s Hospital Colorado, Aurora, CO 80045 USA; 4 Section of Clinical Genetics and Metabolism, Department of Pediatrics, Center for Bioethics and Humanities, University of Colorado Anschutz Medical Campus, Anschutz Medical Campus, Aurora, CO 80045 USA; 5 Department of Molecular Medicine, Department of Neurology, Center for Individualized Medicine, Mayo Clinic, Rochester, MN 55905 USA; 6 Department of Neurology, University of Massachusetts Chan Medical School, Worcester, MA 01605 USA; 7 Institute for Medical Genetics and Applied Genomics, Centre for Rare Diseases, Tübingen University and University Hospital, 72070 Tübingen, Germany; 8 Department of Neurosciences, Division of Pediatric Neurology, Rady Children’s Institute for Genomic Medicine, UC San Diego, La Jolla, CA 92037 USA; 9 Division of Genetics and Genomics, Boston Children’s Hospital, Boston, MA 02115 USA; 10 Division Translational Genomics of Neurodegenerative Diseases, Hertie Institute for Clinical Brain Research, Tübingen, Germany; 11 German Center for Neurodegenerative Diseases (DZNE), Tübingen, Germany; 12 Department of Management, Bentley University, Waltham, MA 02452 USA; 13 Harvard Medical School, Boston, MA 02115 USA; 14 Manton Center for Orphan Disease Research, Boston Children’s Hospital, Boston, MA 02115 USA; 15 Department of Pediatrics, School of Medicine, Section of Neurology, University of Colorado, Aurora, CO 80045 USA

**Keywords:** Individualized medicine, Precision medicine, Rare disease, Ultra-rare disease, Personalized healthcare, Finance, Funding

## Abstract

Individualized medicine has the potential to be a transformative approach to healthcare that tailors medical treatments to the unique makeup of each patient. This report explores the potential of individualized genetic medicines to meet the pressing need for effective treatments for individuals with rare genetic diseases, and funding models to support these treatments. Although recent successes have been achieved, significant administrative and financial challenges remain in implementing individualized treatment trials. This report investigates funding associated with such therapeutics. Funding challenges, key strategic operational factors, potential payor support, historic funding models, and ethical elements ultimately leading to financial support of patient treatment are considered. By addressing these questions, this report aims to provide an overview of the complex issues surrounding individualized medicine, offering insights into balancing its promises with practical and ethical considerations as the field and associated funding models continue to evolve.

## Background

In recent years, therapeutic approaches have broadened from traditional ‘one-size-fits-all’ methodologies to include treatments tailored to patients’ genetic profiles. This transformative approach, known as individualized medicine, enables treatment of common disorders such as cancer, cardiovascular disease, and diabetes, yet al.so offers promise for patients with rare and ultra-rare genetic conditions, a population that has been uniquely underserved [1]. An expanding toolkit of precision therapies—including antisense oligonucleotides (ASOs), CRISPR, small interfering RNA (siRNA), and gene therapies—equips clinicians with the ability to intervene at the genetic level, offering more tailored treatment options.

The emergence of individualized medicine is grounded in significant technological advancements, particularly in clinical genome sequencing. Next generation sequencing technologies such as whole exome sequencing (WES) and whole genome sequencing (WGS) have transformed the discovery and diagnosis of genetic diseases, allowing for more accurate and rapid identification of pathogenic mutations. These technologies have provided the ability to greatly reduce the diagnostic odyssey from years to weeks, and have made early genetic screening both possible and cost-effective.

Advancements in precision diagnostics have not only enhanced our understanding of genetic diseases but have also been accompanied by notable progress in rare disease therapeutics. Historically, the development of treatments for rare and ultra-rare diseases faced significant challenges due to lack of commercial return on investment. Small patient populations proved economically unattractive for pharmaceutical companies to invest in research and development. This prompted the introduction of the Orphan Drug Act in 1983, legislation that offers tax incentives on clinical trials and seven years of market exclusivity for drugs developed as a result. This stimulated investment in the rare disease sector, leading to the development of treatments such as enzyme replacement therapies (ERTs) for conditions like Gaucher disease and Fabry disease, as well as early gene therapies for severe combined immunodeficiency (SCID) and inherited retinal diseases [2–6]. Advances in RNA-based therapeutics also emerged, including antisense oligonucleotides (ASOs) such as Spinraza (nusinersen) for spinal muscular atrophy [7]. Building on this foundation, more recent gene therapy innovations have expanded treatment options for additional rare diseases, including Zolgensma (onasemnogene abeparvovec) for spinal muscular atrophy, Luxturna (voretigene neparvovec) for inherited retinal diseases, and Casgevy (exagamglogene autotemcel) and Lyfgenia (lovotibeglogene autotemcel) for sickle cell disease [8, 9].

The Orphan Drug Act has provided a much needed boost for the most prevalent of the rare genetic diseases, but more than 95% of rare diseases still lack FDA-approved treatments, and developing therapies for all 7,000 known rare diseases could take up to 2000 years if approached sequentially [10, 11]. Building on the foundation established by orphan drugs, there is a growing opportunity to address this challenge. ASOs have emerged as a promising tool due to their ability to be precisely programmed to target a variety of genes. By binding specifically to RNA, ASOs can modulate gene expression, making them a versatile platform for developing treatments for various genetic disorders [12].

In 2018, this potential was realized when the expertise developed for Spinraza was leveraged to develop the first individualized ASO therapy, known as milasen, for a young patient with Batten disease. Spinraza, an ASO, works by binding to the pre-mRNA transcribed from the *SMN2* gene and modifying its splicing to increase the levels of full-length SMN2 mRNA, and thereby levels of SMN protein, which is essential for motor neuron health. Milasen, designed for a distinct genetic mutation, employs the same backbone and chemistry as Spinraza to target a retrotransposon insertion in the *CLN7* gene and restore correct RNA splicing [13]. By adapting the ASO expertise from Spinraza, scientists developed a customized treatment for a single patient’s unique genetic mutation, providing proof of concept for a platform approach towards individualized therapy.

In the years following, this technique was applied to treat other ultra-rare genetic conditions, extending hope to more patients facing previously untreatable diseases [14–16].These cases highlight the emergence of a new paradigm in rare and ultra-rare disease therapeutics wherein development of individualized, or “N-of-1”, therapies leverage the knowledge and infrastructure established through previously approved treatments [17]. Through harnessing multiple therapeutic modalities to target specific genetic mutations, platform technologies offer the collective potential to address previously untreatable conditions. This approach moves beyond traditional drug repurposing or complex chemistry development, allowing for more precise and individualized therapeutic strategies, the potential of which is substantial with estimates suggesting it could someday target as much as 90% of human diseases [18, 19].

This report investigates the funding associated with the development, administration, and overall infrastructure of the first wave of individualized gene targeted therapy trials, examines successful funding mechanisms to date across an international context, and identifies strategies that need to be considered or developed to ensure ongoing access to individualized gene targeted therapies. While the report explores various funding models, it does not specify exact costs, as these vary widely depending on institutional and geographical factors. By addressing these questions, this report aims to provide an overview of the complex issues surrounding individualized medicine, offering insights into balancing its promises with practical and ethical considerations as the field and associating funding models continue to evolve.

## Main text

### Funding and reimbursement challenges in the era of individualized medicine

As scientific opportunities evolve, our regulatory and reimbursement frameworks must also adapt to fully harness the potential of individualized therapies.

In the United States and globally, pharmaceutical companies have long held the key to new drug development pathways. In the US, new therapeutics are developed by pharmaceutical companies who typically secure approval through Biologics License Applications (BLAs) or New Drug Applications (NDAs), which affirm their safety and efficacy and authorize their marketing and distribution. Following this approval, these pharmaceutical companies seek coverage and reimbursement from insurance companies. Sponsors present clinical trial data demonstrating the efficacy and safety of their therapies. These data trigger a detailed negotiation process between the sponsors and insurance providers, the outcome of which determines whether the therapy will receive insurance coverage and the extent of that coverage [20].

In contrast, current individualized therapies are typically originating from investigator-initiated programs and are accessed through expanded access or research programs. These programs allow patients with serious or life-threatening conditions to receive investigational treatments outside of traditional multi-phased clinical trials. Unlike conventional therapies, which are assessed based on data from clinical trials with larger sample sizes, individualized therapies lack such data due to their smaller, more targeted nature. These therapies, given their specialized and limited-market nature, are also not produced nor marketed at traditional scale. Consequently, the reimbursement landscape for these therapies is more complex [20–22].

In the absence of a comprehensive framework to support N-of-1 clinical trials, funding for the development of this first wave of individualized therapies has been pieced together through a combination of public and private investments, grants, and philanthropic donations. Funding for the clinical costs of administering, let al.one development of the therapeutic, is often not covered by insurance and sometimes requires out-of-pocket payments by patients and their families. This fragmented landscape funding poses significant financial challenges, creating barriers to access and highlights the need for more cohesive support structures for these innovative treatments. As N-of-1 clinical trials expand, a comprehensive funding approach will be required to avoid exacerbating disparities within genomic medicine, particularly given the substantial upfront costs of individualized therapies [23, 24]. These costs are often coupled with uncertain benefits that accrue and are assessed over a much greater period of time than a traditional clinical trial. In light of these challenges, an outcome based system has been suggested to provide an avenue for commercial and federal payors to support individualized medicine for its beneficiaries [25]. Other proposals include creating recognized and reimbursable interventional genetics procedures or subscription based models [26].

As the field navigates financial scalability challenges, not-for-profit and philanthropic sources have largely supported the development of individualized medicine. Organizations and initiatives such as the n-Lorem foundation, the Dutch Center for RNA Therapeutics (DCRT), One Mutation, One Medicine (1M1M), and the *N* = 1 Collaborative (N1C) represent examples of organizations operating in this space, providing coordination or support for individualized medicine development. These organizations accept funding from a number of sources such as industry partners, government funding, individual philanthropy and patient advocacy groups as there are few external grant opportunities that support the development costs beyond early stage proof of concept [27]. Furthermore, the reality is that internal funding opportunities often depend on the interests and resources of an investigator’s institution. In light of these challenges, partners have found new and creative ways of contributing to the precision medicine space through donations of time, money, materials, and expertise to researchers and non-profits as a means of supporting these trials [28]. For example, initiatives such as the *N* = 1 Collaborative’s Donated Resource Center demonstrates a coordinated model in which donated goods and services from industry partners support individualized medicine initiatives. This collaborative model helps address the unique challenges of N-of-1 trials by providing critical resources that might otherwise be inaccessible to researchers and small-scale programs. At the same time, renewed interest in a commercially viable platform for development of individualized medicines have created space for companies such as EveryONE Medicines to emerge.

While these mechanisms have facilitated the progression of therapies to the clinical stage, ethical concerns have emerged. Over-reliance on philanthropy and patient-driven funding places the responsibility for financing access to treatments inappropriately upon on patients and their families, as well as raising significant concerns about exacerbating medical disparities and unwanted ‘pay to play’ dynamics [29]. Opinions on patient-driven fundraising are mixed, with some feeling it should be disallowed, while others considering it acceptable when accompanied by rigorous ethical oversight and adherence to established clinical trial standards [30–32]. There is an urgent need to develop alternative funding models that ensure ethical participation and support the equitable development of therapies, as funding sources in individualized medicine have the potential to introduce bias that could influence key aspects including case selection, resource usage, and trial protocol [33].

### Key considerations for funding individualized trials

**Fig. 1:**
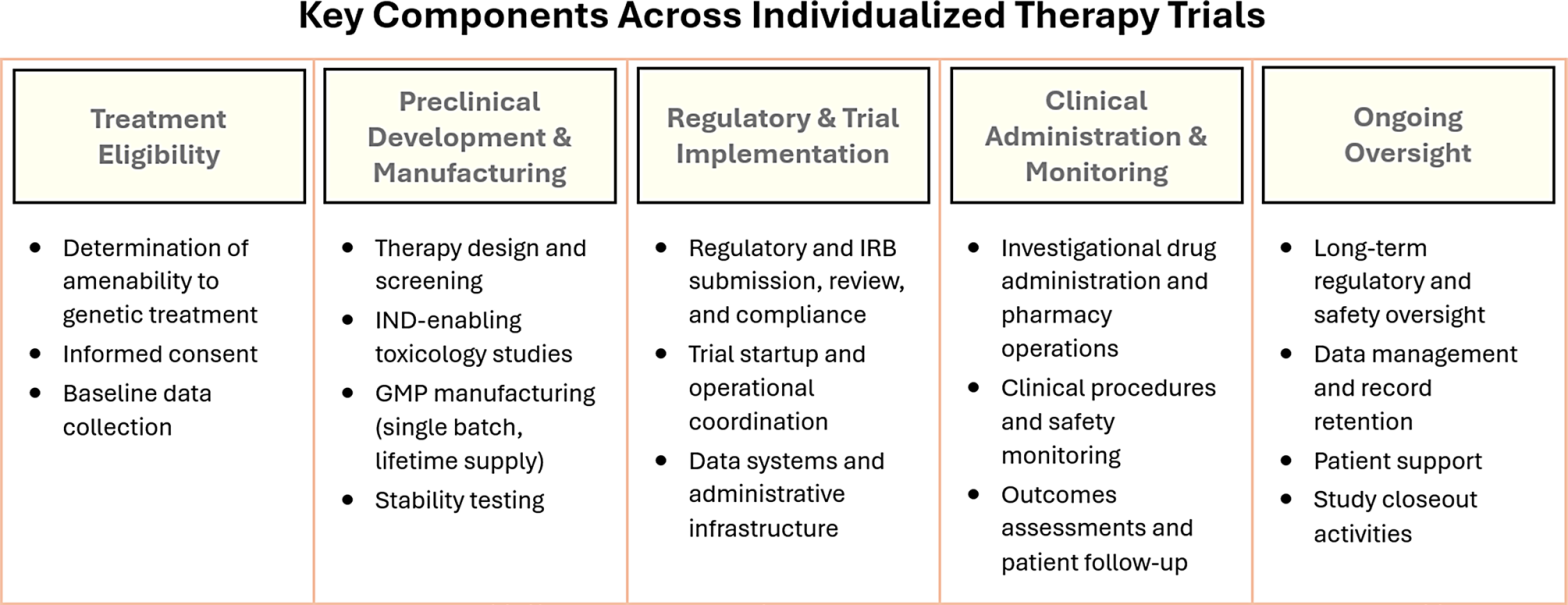
Key components across individual therapy trials. Conceptual overview of scientific, regulatory, clinical, and operational components required to support N-of-1 trials

Funding individualized trials requires careful consideration of institutional support, resource allocation, and funding mechanisms to enable the development, execution, and long-term management of investigational therapies for ultra-rare diseases. The timing, sequencing, and cost of individual components vary widely depending on depending on institutional infrastructure, regulatory pathway, therapeutic modality, and case-specific factors. Institutional support is critical to the development and translation of individualized therapies from initial project planning and patient consent, through execution of N-of-1 trials and transition to clinical care [34]. To date, individualized treatment efforts have been supported by large academic medical institutions with strategic centers or programs with the knowledge, infrastructure, and funding to enable such studies. While most institutions will be unable to self-fund the entirety of an individualized medicine pipeline, there is a minimum requirement of effort and expertise needed to launch and manage patient-specific research prior to acquiring external support if available. Having the expertise, motivation, and sufficient time of the needed individuals to plan, coordinate, and execute each step in a timely fashion to meet the needs of the patient requires a significant level of institutional support and commitment.

Thus, funding mechanisms are required to support institutional implementation across levels, and individual and programmatic grant opportunities enable such discovery and delivery of investigational individualized therapies. Such funding mechanisms are key for specific rare and ultra-rare causal variants not amenable to commercial ASO approaches and for clinical trial participants and patients with most rare genetic variants, an underserved population for whom standard investigational ASO clinical trial approach is not feasible.

Grants supporting genomic-based individualized rare disease therapies spanning preclinical to clinical research may be available from internal academic sources, as well as external state (e.g., CIRM), federal (e.g., NIH, NORD), and nonprofit foundations (e.g., A-T Children’s Project). These may be awarded to individual investigators or structured as programmatic grants to support institutional centers or collaborative networks. In addition, foundations, advocacy groups, and private donors play a critical role in motivating and funding the translation of individualized medicines. Too often, the treatment of patients with life threatening or severely debilitating genetic conditions require rapid timelines, to which traditional grant mechanisms cannot be responsive. Specialized drug development pipelines, infrastructure, and essential financial support through such research funding programs are required to make orphan disease drug development and N-of-1 trials a potential area of research and production.

How is such funding spent? Various aspects need to be considered to adequately support the entire process, from startup to closeout, covering both direct and indirect costs (Table 1).

Subsequent to genetic diagnosis, determination of amenability to genetic treatment must be performed [12]. Additionally, a significant amount of preclinical experimentation and manufacturing need to be performed prior to considering patient treatment. Once a variant has been identified as targetable, many drugs are designed and screened as potential therapies. The lead candidate from this screening is then subject to IND-enabling toxicology studies, as required by US FDA [35]. These studies comprise a large portion of the development costs [26].

In order for an investigational therapy to be available for an individual patient, it must be manufactured in accordance with FDA Guidance [36]. Typically, lifetime supplies are produced in a single batch (typically the smallest batch that can be produced in a GMP facility), though ongoing costs are required to maintain the drug’s safety and quality over time. Stability testing must be performed on the batch at regular, ongoing intervals. Additionally, there must be oversight of the Certificate of Analysis, stability data, and expiration dates of the drug product. Often this ongoing support is provided by an institution’s investigational pharmacy, which must have the resources and expertise to do so. Additional investigational pharmacy costs are described below.

The initial trial implementation phase involves significant preparatory work, which includes clinical research coordinator time and startup fees. Building the electronic data capture system and source documents, performing technology maintenance, and ensuring vendor access training are crucial initial steps. These activities lay the foundation for accurate data collection and compliance with regulatory standards. Additionally, the involvement of a clinical research coordinator is essential for activities including but not limited to managing patient visits, reviewing medical and treatment histories, tracking adverse events, and monitoring vital signs. Only some of these activities may be part of standard of care or office visit fees.

Engaging patients and monitoring their progress is central to the N-of-1 trial methodology. Informed consent and eligibility reviews are critical to ensure that participants meet the study criteria and understand the trial’s purpose and procedures. Regular diary collection (e.g., drug diaries, seizure logs, stool trackers) and administering quality of life questionnaires are necessary for capturing patient-reported outcome measures. These measures are complemented by clinician-reported outcomes, performance-related outcomes (e.g., gait assessments), safety outcomes (e.g., radiological imaging) and molecular treatment-response biomarkers, which can indicate target engagement (Synofzik et al. 2021, Schuele et al. 2024),- [37, 38] Additionally, concomitant medication tracking and the documentation of patient safety events such as serious adverse events, adverse events of special interest, and protocol-required investigations are essential for comprehensive patient monitoring [39].

Administrative costs, including the building and maintenance of systems for data management, CRC administrative tasks (scheduling, electronic data capture entry, visit preparation, post-visit administrative work, patient communication, and site calls), and research nursing startup fees, constitute a significant portion of a related budget. Regulatory compliance is another substantial expense. This includes regulatory administration fees, institutional review board (IRB) submission and annual continuing review fees, IND submissions, protocol amendments, informed consent form updates, budget and contract changes, documentation of staff training, and study closeout or cancellation fees. Translated informed consent forms, whether this be a per-word or per-document fee, should also be accounted for in the budget. Pairing with the translated documents, is the interpreter services utilized for participant contact. This translator will be involved and utilized for any communication with the participant from the consent process through home health or phone communication. Participants may have to travel long distances to be present for in person needs. It is imperative to keep costs in mind for trials that may need to support expenses such as short-term housing, travel costs, and food in order to provide equal opportunities for all who may need the treatment.

Investigational drug services or pharmacy-related preparation may incur startup costs, as well as investigational product dispensation fees, and annual pharmacy fees. Investigational drug service and research pharmacy teams will commonly charge additional fees for storage, record maintenance, and dispensation fees [39]. Clinical coordination expenses include costs for blood draws, nursing administration time, and investigational product administration in various clinical settings (operating room, procedure center, recovery room, post-op observations). Ancillary services such as sleep labs, physical therapy, ophthalmology, and specialty pharmacy services may also incur additional startup fees.

Laboratory costs involve startup fees, sample processing time, and any necessary imaging (ophthalmology, dermatology, etc.). These services are integral to obtaining the clinical and biological data required for the trial. In addition to these safety monitoring fees, additional safety monitoring costs depending on if there are reactions to the treatment or clinical signs that provide a necessity for additional safety testing and monitoring should be factored in.

Annual maintenance fees encompass ongoing study costs, general study supplies, and record storage for regulatory compliance. Miscellaneous fees might include patient stipends, travel reimbursements, annual travel vendor account fees, and medical services resulting from study-related injuries.

**Table 1:** Institutional resources required for individualized therapy

Institutional Resources – A Framework of Key Components for *N*-of-1 Therapeutics	
Personnel	Principal investigator and knowledgeable clinical team
	Basic Science/Translational research team
	Clinical research coordinators and research nurses
	Research protocol specialists
	Regulatory specialists
	Research pharmacists
	Social workers
Infrastructure	Institutional Review Board protocols for consenting to drug development, sample and data sharing, and baseline clinical data collection
	Clinical research and trial units
	Ethics committees
	Drug development capabilities
	Legal support

### Payor support of individualized therapies

While a detailed description of payor support of clinical trials across regions is outside of this report’s scope, two examples are described herein: the United States and Germany. In the United States, while most clinical trial costs are borne by industry sponsors, some trial costs may be billed to payors [40]. The Centers for Medicare and Medicaid Services, in association with the Affordable Care Act, states that payors “may not deny (or limit or impose additional conditions on) the coverage of routine patient costs for items and services furnished in connection with participation in the trial” under Public Health Service Act Section 2709(b) [41]. There exists a great deal of state-to-state statutory heterogeneity for coverage of clinical trial costs associated with phase of trials as well as indication [42]. While some states’ statutes are vague, others are quite explicit [42]. Six states provide no details related to clinical trial coverage. California’s SB-37, however, details oncology clinical trial coverage for Phases I-IV investigations approved by the National Institutes of Health, the U.S. Food and Drug Administration, the U.S. Department of Defense, or the Department of Veterans Affairs [43]. This lack of state-to-state consistency presents a complex environment for translating an approach to payor support of N-of-1 clinical trials across institutions and geographies in the United States.

The inconsistency in state laws complicates clinical trial coverage and is further compounded by a broader challenge: the misalignment of traditional payor support structures with the unique needs of N-of-1 clinical trials. Payor support is typically associated with procedures and testing but not with therapeutic development and production, whose cost is borne by a sponsor. This nuance is particularly important when considering bespoke therapeutics aimed to treat individual patients. Currently, there is no mechanism by which the cost of developing a therapeutic can be recovered upon approval to treat. Secondarily, most N-of-1 clinical trials have relied upon nonprofit organizations to develop or support the development of treatments. In most instances, a drug developer donated time and resources to produce a therapeutic for an individual.

### Payor support case example: United States

One anecdotal example from the U.S. illustrates how insurance coverage was successfully obtained for procedures and safety measures, though not drug development costs, associated with an ASO therapeutic developed for an individual patient [44]. In this case, the treating institution submitted a prior authorization request approximately 3 weeks prior to implementation of treatment, after determining the appropriate procedural terminology codes. Clinical notes and prior authorization requests supporting treatment were also submitted to support the reasoning for proceeding with treatment. Ultimately, the insurer agreed that no prior authorization was required as these procedures were based on medical necessity with no exclusions nor limitations. While this was an ideal outcome for the patient, it should be noted that the treating institution internally approved a waiver of any expenses not covered by insurance. To ensure that this was the case, the patient’s account was flagged and reviewed extensively prior to any bills being sent to the patient. This example suggests that with consistent application of clear documentation and alignment with existing billing structures, a framework for payor coverage of procedures and safety measures (while still not for drug development costs) of individualized therapies may be possible as a standard practice.

### Policy framework example: Germany

In contrast to the individual case example presented above, this section describes the national policy and reimbursement framework in Germany. In Europe and thus also Germany, the regulatory framework for treating individual patients with investigational individualized ASO treatments is the *named patient* use of these experimental medicines [37]. Under this model, statutory health insurance (SHI) covers the costs of the medical standard, but not the costs that serve to gain knowledge associated with applying this standard and also not the costs to generate a medical standard in the first place. However, a *named patient* treatment can meet the medical standard owed by the SHI if the success of a measure explicitly intended to treat the patient is still subject to uncertainty due to existing evidence gaps and is therefore tested to see whether the patient responds individually to the treatment.

Funding of individualized treatment by the SHI fundamentally requires that the treatment is covered by the scope of benefits provided by the SHI scheme. Under this scope, patients are entitled to treatment that corresponds to the generally recognized state of medical knowledge and the principle of cost-effectiveness, taking into account scientific progress (Section 2 (1) sentence 3; 12 (1); 27 SGB V) [45]. As a rule, entitlement to benefits presupposes that the treatment is approved for the area of application in which it is used (on-label); off-label use is only permitted in strictly limited exceptional cases.

For outpatient care, the use of new treatment methods is generally prohibited until the Joint Federal Committee (J-FC) includes them in the SHI benefits catalog following their assessment (Section 135 [1] SGB V) [45]. This reservation of permission does not apply to treatment methods that are used as part of inpatient care. Methods on which the J-FC has not yet made a decision may be used in the context of hospital treatment and claimed by insured persons *“if they offer the potential of a necessary treatment alternative and their use is in accordance with the rules of medical practice*,* i.e. in particular if they are medically indicated and necessary”* (Section 137c [3] sentence 1 SGB V). Even if the above conditions are not met, the patient may still be entitled to benefits. This means that *“insured persons with a life-threatening or regularly fatal illness or with an illness that is at least comparable in terms of value*,* for which a generally recognized benefit corresponding to the medical standard is not available*,*… can also claim a … different benefit if there is a not very remote prospect of a cure or a noticeable positive effect on the course of the illness* ” (Section 2 (1a) SGB V) [45]. This regulation, which goes back to the Nikolaus decision of the Federal Constitutional Court (BVerfG, decision of December 6, 2005–1 BvR 347/98), significantly lowers the evidence requirements in the area of life-threatening illnesses [46]. However, the case law specifies the criteria of life-threateningness or a regularly fatal course restrictively. Accordingly, there must always be an emergency-like situation with a very limited lifespan (< 5 years). Furthermore, the Federal Social Court has ruled that the existing evidence requirements for treatment methods for inclusion in the SHI benefits catalog do not apply if scientifically sound statements are not possible because the disease occurs “*only extremely rarely worldwide*” and can therefore neither be systematically researched nor systematically treated within a national or international framework [46].

The authors are not aware of any practical cases in which this case law has led to a claim for benefits. However, for individualized treatment, which concerns the treatment of diseases in which only one patient or one or a few families are affected, it might be that an entitlement to benefits can be well justified on the basis of this rarity case law, so long as a favorable risk-to-benefit ratio exists. Such an entitlement to benefits always presupposes that there is no treatment option that meets the medical standard in the specific individual case. If a patient with statutory health insurance is entitled to treatment according to the criteria described above, this entitlement must be fulfilled by the health insurance fund. As the treatment is provided on an inpatient basis, the health insurance fund fulfils its obligation by transferring the fees regulated in Sect. “Conclusions” Krankenhausentgeltgesetz, in particular the revenue budget - determined via the diagnostic related group system - as well as individually agreed hospital fees, such as the so-called “novel examination and treatment methods” fees [47].

This can lead to successful insurance coverage for the procedures and safety measures associated with an ASO therapeutic developed for an individual patient, thus in the outcome very similar to the example from the USA above. Yet, similar to the US example, it still does not cover the costs for the drug itself nor for the development of the drug- which are by far the largest treatment cost of such individualized therapies. Funding models that enable recovery of individualized therapeutic development costs generally require sufficient evidence to secure formal regulatory approval under German law, which investigational individualized therapies often remain without.

In the theoretical scenario where a patient is entitled to an individualized treatment, but standard fees covered by insurance do not adequately cover the cost of the treatment, the financial shortfall is assumed by the hospital. In such cases, the hospital fulfills the patient’s entitlement to treatment and submits an application to the health insurance fund. In practice, the health insurance fund may approve additional coverage on a case-by case basis, including when a new treatment is not yet covered by the diagnostic related group system or is designated as a novel examination and treatment method. In situations of urgent medical need, SHI may even be obligated to approve such coverage.

Payor coverage for N-of-1 clinical trials is a complex and challenging landscape, shaped by legal and regulatory inconsistencies both within and between countries. The examples from the U.S. and Germany highlight the difficulties of navigating a fragmented system, where varying laws, payer structures, and healthcare policies create a web of uncertainty. These challenges underscore the urgent need for more harmonized and adaptive approaches to payor support for individualized medical innovations.

### A survey of funding models: Past strategies for N-of-1 clinical trials

**Fig. 2:**
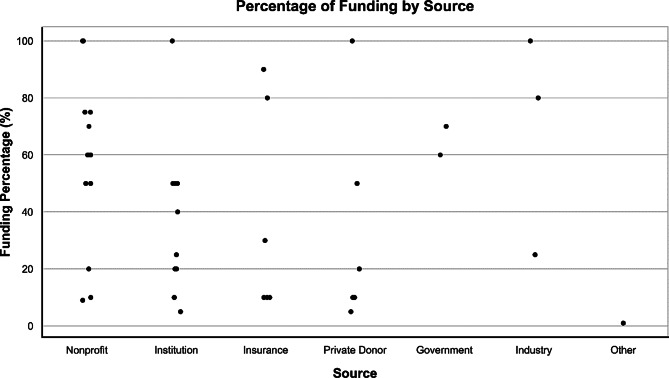
Percentage of funding supporting individualized therapy by funding source

With the challenges described in mind, and having reviewed the key considerations in funding individualized medicine trials, we present here results of a survey conducted to better understand various funding approaches used to support N-of-1 clinical trials. These funding mechanisms have either been suggested for funding N-of-1 trials, have been seen to date, or are used in reimbursement in other similar situations (e.g. precision medicine). We will further examine these mechanisms through the results of our survey and additional case examples, as an aim of this report is to ascertain and disseminate how successful therapies have been funded historically to better conceptualize models in future state (Table 2). All relevant data are presented in their entirety in Table 2; Fig. 1, and Fig. 2.

**Fig. 3:**
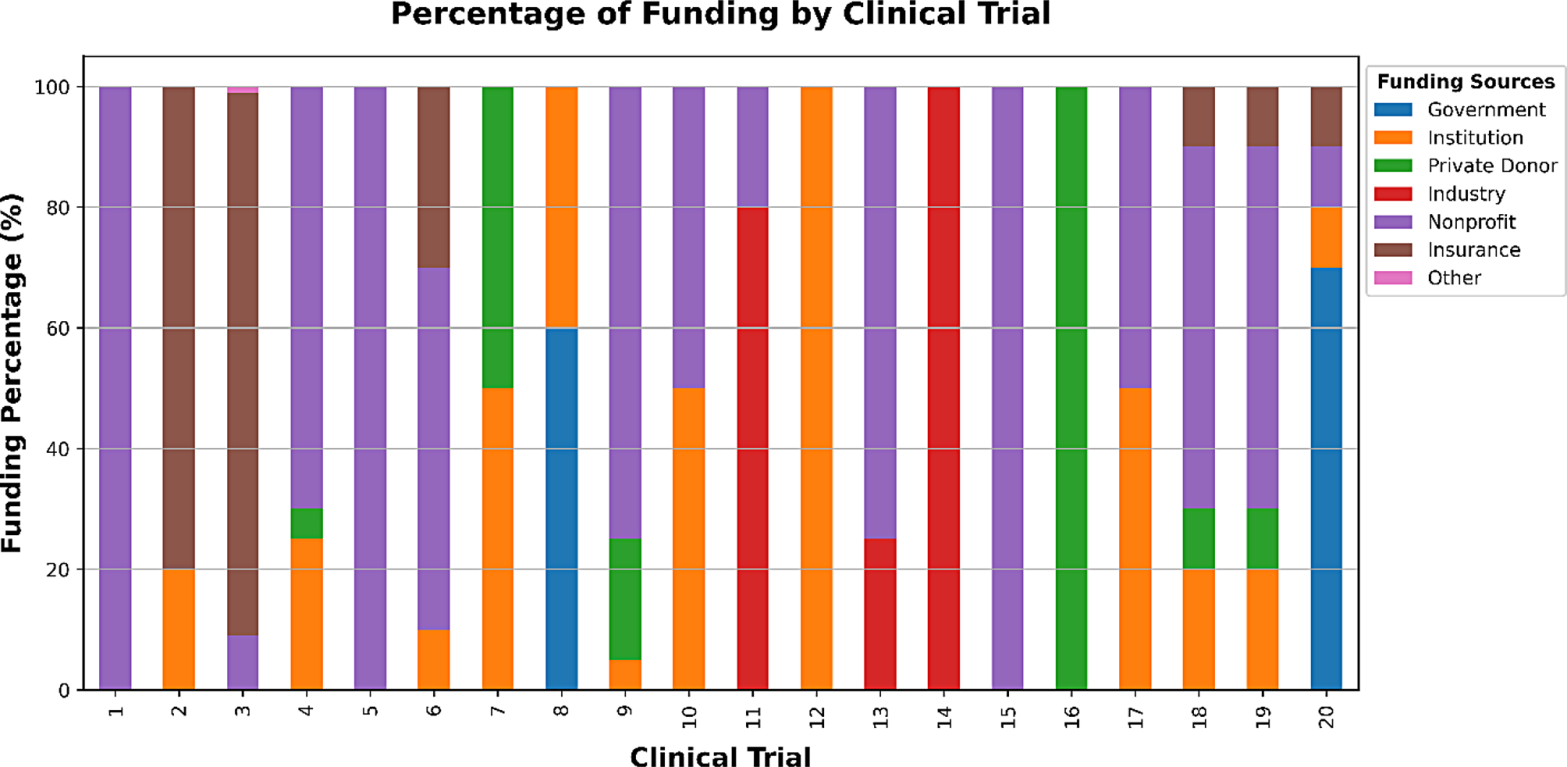
Percentage of funding supporting individualized therapy by discrete therapy

Invited respondents were identified by the N1C’s network of members. The proportion of funding from sources including government, treating institutions, private donors, industry, nonprofit organizations/foundations, and insurance was determined. Respondents were also allowed to note the proportion of funds from sources other than those noted above. Respondents were additionally asked to summarize the funding model utilized including advantages, disadvantages, variability between trials, and any other notes of interest. In total, twenty valid responses were received from countries including the United States, Germany, and the Netherlands. This survey investigation was deemed non-human subjects research by the Colorado Multiple Institutional Review Board (23-1605).

Visual inspection of Figs. 2  and 3 reveals great variability in the funding of N-of-1 clinical trials. Notably, only five of the twenty clinical trials were supported by single financial mechanisms while the remainder used a combination of funding sources. Those trials supported by single financial mechanisms displayed great variability in the funding sources utilized, in that the five studies used four discrete mechanisms. Of those clinical trials supported by combinations of funding sources, the majority included nonprofit funds and financial support of their home institutions. Six trials were supported by insurance, with some procedures and assessments being performed as standard of care.

Several themes emerged when respondents were asked to remark on the advantages of their funding models. Several were supported by nonprofit groups, such as n-Lorem, which bore the cost of treatment development and production. Additionally reported were distinct examples of significant governments and payor support of specific bespoke treatments. Thirdly, support of N-of-1 clinical trials by patient advocacy organizations was reported to better ensure access to treatment for patients with specific conditions.

The disadvantages associated with the funding models noted by respondents were nearly universal and focused on two domains: sustainability and repeatability. 75% of these clinical trials were financially supported by several sources of funds. Replicating this would be difficult at the same institution, much less at different institutions. Twelve of the twenty trials were also financially supported by their home institution. With finite resources and increasing demand, respondents noted concerns regarding institutions’ ability to support new treatments when developed. Additionally, patients and families may be financially burdened by costs ancillary to treatment noted by the single “Other” response to the survey, which represented accommodation costs associated with travel for treatment. A final broad concern noted across responses was funding of personnel support. Much of the providers’ time supporting treatment was borne by the institution, and several responses indicated that this was in addition to the providers’ normal workload. Additional support was also required for research specific activities not associated with typical clinical care. Such activities included regulatory submission and maintenance, research contracting, financial management, and study coordination. The complexity in all of these activities is frequently increased and emphasized for N-of-1 clinical trials, particularly during the study start up process, when compared to more traditional and better understood study models.

The great variability in the results of this survey and the limited sample size preclude analyses using more sophisticated methods than descriptive statistics. Furthermore, we believe that these limitations mask underlying differences based on geography. The results of this survey support our presumption that there exists no common funding model for funding N-of-1 clinical trials. This finding demonstrates the necessity for greater prospective structural support of such treatments. We believe that this such structure would provide greater sustainability of each trial to better ensure participant safety. Furthermore, greater commonality in support mechanisms would ensure the feasibility of implementing additional trials to treat additional patients.

**Table 2:** Overview of percentage of individualized therapy supported by funding source type

Funding Source	Clinical Trials (*n*)	Mean Proportion (%)
Government	2	65
Institution	12	33
Private Donor	6	33
Industry	3	68
Nonprofit	14	60
Insurance	6	39
Other	1	1

### Learning from established medical practice models

As the practice of individualized medicines emerges, comparisons to established medical practice models including oncology, transplantation, and surgery offer useful reference points for understanding how complex, high-cost interventions have been historically developed and sustained. Oncology provides a particularly relevant reference in this context given its high preclinical investment and development risk.

The development of treatments for both cancer and ultra-rare diseases shares key similarities, particularly in how risk and uncertainty are managed. Both fields also operate under development conditions characterized by uncertainty and upfront technical investment. In the case of rare diseases, the limited patient populations for clinical trials, coupled with incomplete knowledge of disease mechanisms, heightens translational risk. Similarly, cancer therapies, particularly in the context of precision oncology, face challenges of tumor heterogeneity, resistance mechanisms, and individual patient variability [48].

While both fields pursue targeted, mechanism-driven approaches, oncology development efforts are supported by established translational pipelines and funding structures capable of absorbing high investment. In contrast, comparable support structures remain limited for individualized therapeutic development efforts, where similar technical demands are incurred without the benefit of reimbursement or scale.

This structural asymmetry is reflected in national research investment patterns. Funding appropriated by the National Institutes of Health (NIH) in 2019 highlights a striking disparity: $5.74 billion was allocated for oncology research, compared to just $38 million for rare disease research—a 151-fold difference [49].

### Ethical considerations in the funding of N-of-1 therapies

A significant concern regarding individualized therapies is the potential to exacerbate existing health disparities in genomic medicine. Studies have demonstrated that referral for genetic counseling or genetic testing vary significantly by race/ethnicity and geographic location [50, 51]. Furthermore, the majority of genomic data in existing databases originates from individuals with European ancestry and may limit the benefit of genomic testing for many individuals with human ancestries that are not well represented [52, 53]. Ensuring access to, and increasing the benefit of, genetic testing for all is critical to ensuring equitable access to individualized genetic therapies.

As detailed, patient-driven crowdfunding has played a significant role in financing several individualized therapy programs. While laudable, this approach has the potential to amplify standing inequities related to access to medical care, as crowdfunding may favor patients with compelling personal narratives over those who may have greater medical need but less visibility [54]. Studies have also demonstrated that factors such as gender, race/ethnicity, and geographic location can negatively impact fundraising [55–57]. Non-profit groups have been established to accept funding from various sources and can help negate some of the concerns that are associated with individual crowdfunding. However, the potential for bias remains a concern, particularly as patient-driven funding has the potential to motivate academic investigators. It is important to consider appropriate oversight about the scientific merits of any given therapy to ensure that funding decisions are based on medical need and potential benefit.

The efforts of non-profit organizations, families, philanthropic donors, and academic investigators to make individualized therapies are commendable. However, additional funding mechanisms are necessary to ensure that treatment decisions are grounded in medical need and potential benefits of therapy. In doing so, we can ensure that individualized therapies become accessible to all individuals, regardless of their financial resources or personal circumstances.

## Conclusions

Individualized medicine has emerged as a field able to treat rare and ultra-rare conditions. A funding model supporting the development of, and patient treatment with, individualized therapeutics is necessary for progress in ameliorating these conditions. Such funding is necessary for the identification of amenable patients, preclinical development, therapeutic production, patient dosing, monitoring, and the administrative burden of the associated activities. Previous individualized treatments have relied upon government, treating institutions, private donors, industry, nonprofit organizations/foundations, and insurance resources. The distribution of resources used varies drastically on a case-by-case basis. As a comparison, the model for oncology research funding is useful, however a substantial amount of difference exists in the treatment of other conditions. Additionally, ethical concerns exist related to the equity of treatment access. A model for funding individualized therapeutics must be established for the treatment of rare and ultra-rare conditions.

## Data Availability

All data collected are presented in this report as tables and/or figures.
